# Nanoparticles for the Treatment of Inner Ear Infections

**DOI:** 10.3390/nano11051311

**Published:** 2021-05-17

**Authors:** Dan Cristian Gheorghe, Adelina-Gabriela Niculescu, Alexandra Cătălina Bîrcă, Alexandru Mihai Grumezescu

**Affiliations:** 1 “Carol Davila” University of Medicine and Pharmacy, 050474 Bucharest, Romania; gheorghe.dancristian@gmail.com; 2 “M.S. Curie” Clinical Emergency Hospital for Children, 050474 Bucharest, Romania; 3 Faculty of Engineering in Foreign Languages, University Politehnica of Bucharest, 060042 Bucharest, Romania; niculescu.adelina19@gmail.com; 4 Faculty of Applied Chemistry and Materials Science, University Politehnica of Bucharest, 060042 Bucharest, Romania; ada_birca@yahoo.com; 5 Research Institute of the University of Bucharest—ICUB, University of Bucharest, 050657 Bucharest, Romania

**Keywords:** inner ear, infections treatment, antimicrobial nanoparticles, drug-delivery systems, potential side effects

## Abstract

The inner ear is sensitive to various infections of viral, bacterial, or fungal origin, which, if left untreated, may lead to hearing loss or progress through the temporal bone and cause intracranial infectious complications. Due to its isolated location, the inner ear is difficult to treat, imposing an acute need for improving current therapeutic approaches. A solution for enhancing antimicrobial treatment performance is the use of nanoparticles. Different inorganic, lipidic, and polymeric-based such particles have been designed, tested, and proven successful in the controlled delivery of medication, improving drug internalization by the targeted cells while reducing the systemic side effects. This paper makes a general presentation of common inner ear infections and therapeutics administration routes, further focusing on newly developed nanoparticle-mediated treatments.

## 1. Introduction

Inner ear disorders affect an important portion of the world population, deafness being the most common sensory impairment worldwide [1,2,3]. A significant share of this burden is caused by sensorineural hearing loss (SNHL), originating from cochlear defects [3]. Some of these defects have been reportedly caused by various congenital and acquired inner ear infections [4,5,6,7,8].

Hence, ear disorders’ prevalence represents an acute incentive and opportunity towards improving therapeutic interventions [9,10]. Specifically, the small size, limited accessibility, and high vulnerability of the inner ear pose certain difficulties, narrowing down treatment options [11]. Currently used methods, such as systemic delivery, intratympanic injection, and direct inner ear drug delivery, often face challenges in terms of efficacy and invasiveness [1,9]. 

The anatomical and physiological barriers of the ear coupled with the low long-term stability of drug molecules are the main factors that hinder drug penetration and permeation, resulting in sub-therapeutic concentrations at the required site [12]. To overcome the issues of conventional drug-administration, nanotechnology is receiving increasing attention in the field of auditory science [13]. Particularly, nanoparticle-based systems have been proven advantageous in controlled and targeted drug-release, protecting pharmacological formulations up to the desired site, facilitating transmembrane transport, increasing cell uptake, and reducing required doses and side-effects [14].

In this respect, the present paper aims to describe the most common inner ear infections, the causes behind these affections, and current medication administration routes. Moreover, the newest advancements in nanoparticle-mediated treatments are presented in detail, with a special focus on their safety and efficacy.

## 2. Inner Ear Infections

The inner ear is sensitive to infections that may produce permanent SNHL and vestibular dysfunction [15,16]. One criterion for distinguishing between different inner ear infections concerns the affected structures. From this point of view, there are two main possibilities: labyrinthitis and vestibular neuronitis [17].

As its name implies, labyrinthitis is an infection located in the membranous labyrinth. This structure is usually affected by bacterial translocation into the inner ear [17], causing vertigo, nausea, vomiting, tinnitus, and even hearing impairment or hearing loss [18]. The inflammation can result through two different mechanisms. Inflammation can be a secondary manifestation caused by bacterial toxins and/or host cytokines and inflammatory mediators, producing serous labyrinthitis; or it can be caused directly by the bacteria, leading to suppurative labyrinthitis [18]. Particularly, the latter form of labyrinthitis can have severe complications; due to the proximity to the central nervous system, it can progress to intracranial infectious complications, requiring prompt treatment [15,17,19].

Vestibular neuronitis (or neuritis) is often used as a synonym for labyrinthitis, being usually assimilated as a viral infection rather than bacterial [17,20]. However, this term can only be used when just the vestibular portion of the eighth cranial nerve is involved [18,20]. This infection results in a sudden unilateral loss of peripheral vestibular function manifested in the acute phase through prolonged rotational vertigo, nausea, vomiting, postural imbalance, and spontaneous nystagmus [21,22]. As the cause is ultimately due to a virus, this infection’s management is symptomatic [17].

**Figure 1:**
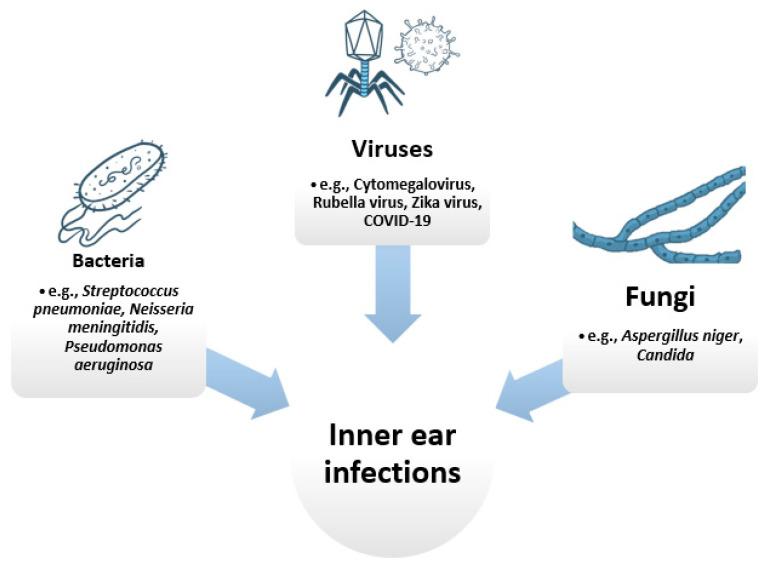
Main categories of pathogens causing inner ear infections. Created based on information from literature References [23,24,25,26,27,28,29,30].

Inner ear infections can also be classified depending on their causing pathogen. In this respect, three categories can be distinguished: viral, bacterial, and fungal infections (Figure 1).

### 2.1. Viral Infections

Viral infections are assumed to play a direct or indirect role in the causation of several inner ear disorders [31]. Viruses can either directly affect inner ear structures; they can induce inflammation that further produces damages; or they can increase the susceptibility to bacterial or fungal infections, eventually leading to hearing loss [26].

Several viral infections that can be congenitally acquired can produce SNHL [32]. A leading cause of non-inherited SNHL is congenital cytomegalovirus infection [27,33,34,35]. This DNA virus is a member of the *Herpesviridae* family and is widely spread in the community. Subclinical infection with cytomegalovirus can affect all bodily organs, including the middle and the inner ear [36]. When an inner ear infection occurs, the marginal cell layer of the stria vascularis is always infected, followed by infection progression into the Reissner’s membrane [27]. This further leads to alteration of sensory structures by dysregulation in ion homeostasis, particularly in the potassium circulation [37]. 

Rubella (or German measles) is a contagious viral infection that most frequently occurs in the fetus during pregnancy, being one of the most common causes of hearing loss in newborns. During the sixth to twelve weeks of pregnancy, the inner ear was reported to be most susceptible to damage. SNHL in babies infected with the Rubella virus is associated with hemorrhagic damage of the organ of Corti. Interruption in further development of different parts of the inner ear and auditory nerve is also reported [15,28,38,39].

A relation between Zika virus infection and hearing loss was also reported in both infants and adults. Zika virus was found to produce a graded distribution of cellular damage in the cochlea, with the greatest damage in the apex, in a manner similar to cytomegalovirus infection [32,40,41].

Recently, it has been noticed that COVID-19 infection could have deleterious effects on the hair cells of the cochlea, despite being asymptomatic. However, further research is needed for properly understanding the mechanism of these effects [26,42,43].

### 2.2. Bacterial Infections

SNHL can also result as a complication and sequela of bacterial infections, such as meningitis [29,44]. The most common causes of bacterial meningitis in the first 90 days of life are Group B *Streptococcus* and *Escherichia coli*, while, in children, SNHL is more frequently associated with meningitis caused by *Streptococcus pneumoniae* or *Neisseria meningitidis* [29]. The released inflammation by-products (e.g., nitric oxide, superoxide, peroxynitrite) contribute to the disruption of the blood labyrinth barrier, inducing a cytotoxic effect on the cochlea. The inner ear can also be damaged through vascular occlusion, which may further lead to cochlear hypoxia and ischemia, and neural damage [44].

Another bacterial infection is otitis media. Generally caused by microorganisms like *Pseudomonas aeruginosa*, *Staphylococcus aureus*, *Proteus mirabilis*, *Klebsiella pneumonia* and *Escherichia coli*, this infection is mainly located in the middle ear [45,46]. Out of the enumerated pathogens, *Pseudomonas aeruginosa* is one of the most common bacteria to produce chronic suppurative otitis media and reach perilymph by entering through the round window [30,47]. Moreover, recurring otitis media can destroy ear structures such as small bones, seventh cranial nerve or inner ear, leading to permanent hearing loss [45].

### 2.3. Fungal Infections

Compared to other infection sources, fungal infections of the inner ear have only rarely been reported [48]. These pathogens usually affect the auditory canal and middle ear arc, often being regarded as harmless saprophytic growth [49]. However, in immunocompromised individuals or patients undergoing long-term antibiotic treatment, such infections may become clinically significant and extend to inner structures [48,49]. Other factors in otomycotic infection progression are humidity, moisture, bathing, and self-hygiene. In swimmers and divers, the external ear canal and tympanic membrane can be infected, and, because of water pressure, pathogens can reach further to the middle and inner ear. The main pathogens responsible for otomycosis are *Aspergillus niger* and *Candida* [25,50].

## 3. Administration Routes

**Figure 2:**
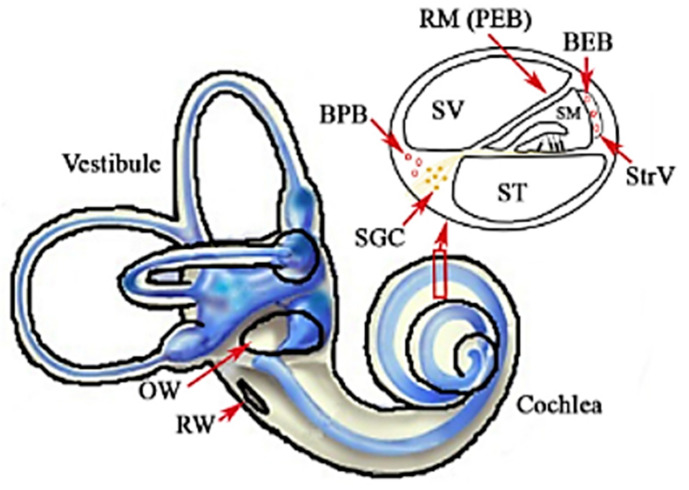
Inner ear barriers: middle-inner ear barriers—the oval window (OW) and the round window (RW); blood-inner ear barriers: the blood-endolymph barrier (BEB) and the blood-perilymph barrier (BPB); the perilymph-endolymph barrier (PEB)—Reissner’s membrane (RM). Other abbreviations: SGC—spiral ganglion cell; SM—scala media; ST—scala tympani; StrV—stria vascularis; SV—scala vestibuli. Reprinted from an open-access source [57].

Inner ear infections are challenging to treat due to their isolated location. Placed in the temporal bone, the inner ear is protected by many anatomical and physiological barriers, which hinder therapeutics’ delivery (Figure 2) [14,51,52,53,54,55,56].

When the inner ear gets infected, aggressive treatment is required to try to prevent complete and permanent loss of cochleovestibular function and avoid spreading the infection to intracranial structures. Generally, the treatment includes administering anti-infective and anti-inflammatory medications, surgical intervention for draining abscesses, and supportive care for the associated symptoms [10,15]. Nonetheless, the treatment’s efficacy and safety are highly dependent on inner ear drug delivery systems [58].

Nowadays, the first-line approach for treating inner ear disorders is the systemic delivery of medication [10,12]. It involves the oral, intravenous, or intramuscular administration of therapeutics that are further distributed throughout the entire organism, despite being needed only in a small body part [9,12]. This administration route has two main drawbacks: it leads to systemic side effects and limits the drug concentration reaching the target site [9]. To avoid these issues, local drug delivery started to be utilized as an alternative [10].

The most commonly used local administration method is intratympanic drug injection [59,60,61]. When using this technique, the drug enters the middle ear cavity and must remain there for a sufficient time to pass through the round or oval window and reach the inner ear [61,62,63]. This administration route allows higher concentrations of medicines at the target site without metabolism “first-pass” [11]. However, drugs do not always stay in contact long enough with the two windows and are discharged to the Eustachian tube before reaching the inner ear in sufficient amount [62].

Another approach is to deliver the necessary drugs directly into the inner ear cavity. The method supposes passing a needle through either the round window or oval window and discharging the drug load into the cochlea or vestibule, respectively [58]. Alternatively, the drug can be released by a cochlear implant [64,65,66,67], osmotic mini-pumps [68,69,70,71], or through reciprocating perfusion systems [72,73,74]. This technique significantly increases drug bioavailability in the inner ear, having the highest efficiency among all administration possibilities [61]. Nonetheless, this is a highly invasive approach, requiring surgical intervention [51].

**Figure 3:**
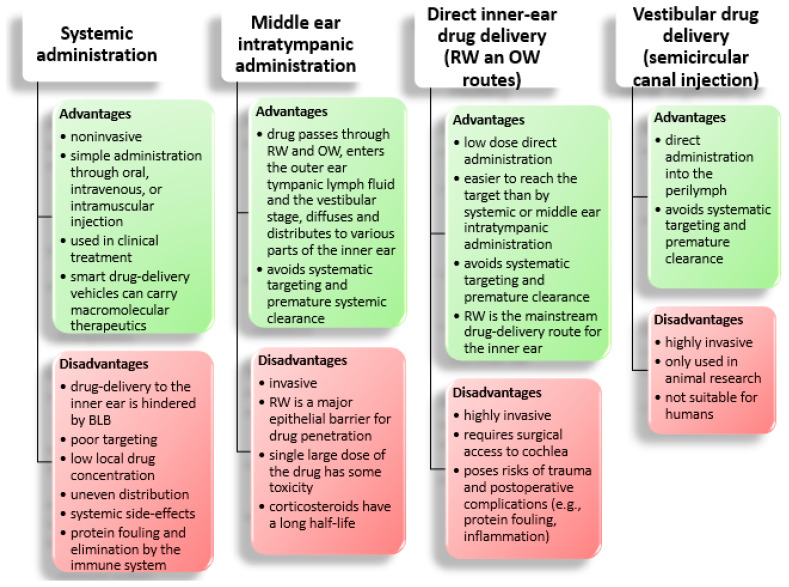
Comparison of different inner ear administration routes. Abbreviations: BLB—blood-labyrinth barrier, RW—round window, OW—oval window. Created based on information from literature References [14,51,54,56,75].

A comparison of the inner ear administration routes is provided in Figure 3.

## 4. Nanoparticles-Mediated Treatment

Other delivery approaches had to be explored to overcome the limitations of traditional drug administration methods [14,76]. Various inner ear delivery systems (e.g., solid foams, hydrogels, nanoscale structures) are investigated to improve local effectiveness and reduce systemic adverse effects [11,77].

One of the most promising solutions is to include nanoparticles (NPs) in the therapeutic strategy [1,13]. Their small sizes (<1 μm) coupled with their inherent physical, chemical, and biological properties render nanoparticulate systems suitable for crossing barriers and efficiently treat inner ear infectious disorders [9,12,55]. In recent years, rather than simply investigating their permeation into the inner ear, research was focused on loading drugs into/onto NPs and transferring them to the inner ear to observe functional changes [62].

Delivering medication via NPs is considered advantageous, especially in terms of drug stabilization for controlled release and surface modification for specific targeting [12,58,78,79]. After administration into the middle ear, loaded-NPs diffuse through the round window membrane, facilitating the freed-drug passage into the cochlea [9,77]. NPs compensate drug properties in terms of low solubility, degradation, and short half-life, this approach reportedly leading to improved transmembrane transport, increased uptake and internalization of drugs by targeted cells (e.g., hair cells, spiral ganglion neurons, pathogen entities), reduced required doses, and subsequent diminished side effects [14,55,62,80,81].

**Figure 4:**
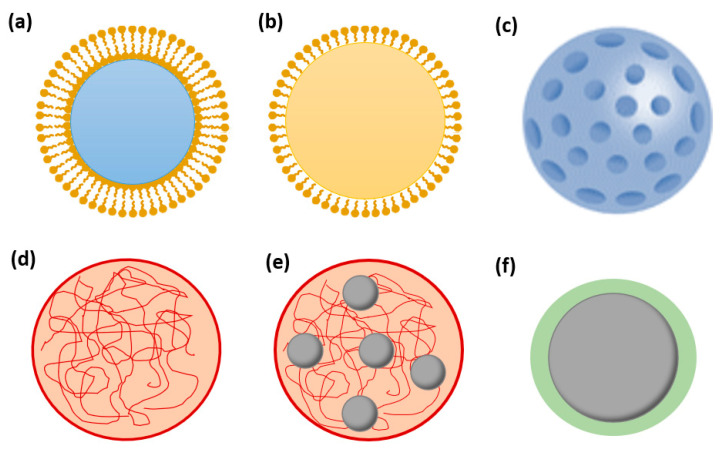
Schematic representation of various nanoparticles: (**a**) liposome; (**b**) lipid core nanoparticle; (**c**) ceramic nanoparticle; (**d**) polymeric nanoparticle; (**e**) superparamagnetic iron oxide NP (SPION)-loaded polymeric nanoparticle; (**f**) coated SPION. Created based on information from literature References [11,12,13,51,83,84,85].

To achieve such results, various NP-based delivery systems are under development (Figure 4) [1]. Inorganic, lipid, and polymeric materials can be employed to fabricate nanocarriers for hydrophilic and/or hydrophobic drugs to be released in a targeted and controlled manner [12,77,80,81,82].

### 4.1. Inorganic Nanoparticles

Metal-based NPs with inherent antimicrobial activity are one of the most extensively researched materials [83,86]. Silver nanoparticles (AgNPs) are of special interest against infections, exhibiting strong activities in antibacterial, antiviral, and antifungal studies [87,88,89,90,91]. AgNPs can physically interact with various bacterial cells’ surface, damage the cell membranes, and produce structural changes that render these pathogens more permeable [89]. Specifically, AgNPs can reach the inner ear in a dose-dependent manner after intratympanic administration and destroy pathogens either alone or in combination with various antibiotic formulations [89,92]. This is a highly advantageous ability against multi-drug resistant bacteria, such as *P. aeruginosa*, overcoming the drawbacks of free antibiotics and eliminating the microorganisms with high efficacy in the ear therapy [87].

Gold nanoparticles (AuNPs) can also carry hydrophilic and hydrophobic molecules, being also researched for imaging applications [93,94]. There are no studies in the literature on AuNPs delivery to the inner ear yet [95], but these nanoparticles have been tested as candidates for inner ear contrast agents [96]. Despite not obtaining a significant imaging enhancement, the study reported successful localization of AuNPs in cochlear cells, which is an encouraging result for future tests. Besides, biomolecules, polymers, and proteins can be used to improve the therapeutic properties of AuNPs, such as their biocompatibility, biodistribution, stability, and half-life [93]. For instance, AuNPs functionalized with 5-fluorouracil showed bactericidal effects against Gram-negative bacteria and antifungal activity against *Aspergillus fumigates* and *Aspergillus niger* [97]. Therefore, it can be expected that combinations of AuNPs and other substances would soon be developed for inner ear drug delivery platforms.

Superparamagnetic iron oxide nanoparticles (SPIONs) are another promising strategy as they can be magnetically guided across the round window and precisely reach the targeted inner ear structures [13,56,59]. Moreover, their relatively simple synthesis, low toxicity, intrinsic antimicrobial activity, and functionalization ability are very important properties for designing effective biocompatible nanoplatforms [98]. SPIONs cannot encapsulate any substance, but they can be loaded into polymeric nanoparticles or coated with the needed drug. For inner ear drug delivery, SPIONs have been tested in combination with PLGA, chitosan, silica, and dextran [13,51,56]. Such nanocomposites can significantly enhance antibiotics’ activity against both Gram-positive and Gram-negative bacteria, being a helpful tool in treating multi-drug resistant pathogen strains infections [99,100,101].

Other metal-oxide based NPs that have been proven effective against inner ear-related infectious diseases are titanium dioxide (photocatalytic effect against fungi and bacteria) [90], zinc oxide (strong antimicrobial activity against *Staphylococcus aureus*, *Escherichia coli*, *Klebsiella pneumoniae*, and *Pseudomonas aeruginosa*; anti-*Candida albicans* properties) [102,103], aluminum oxide (strong growth-inhibitory effect on *E. coli*) [104], silver oxide (reasonable bactericidal activity against *E. coli*, *P. aeruginosa*, and *S. aureus*) [104], copper oxide (anti-*C. albicans* properties) [103], calcium oxide (bactericidal activity against *E. coli* and *S. aureus*) [97], zirconium oxide (potential inhibitory action against *P. aeruginosa* and *S. aureus* and good inhibition against *E. coli*) [105].

Silica nanoparticles are also attractive for carrying medicine due to their commercial availability, narrow particle size distribution, and biodegradability under physiological conditions [106,107]. Particularly, mesoporous silica nanoparticles (MSNs) can be employed to manufacture controlled-release antimicrobial platforms by encapsulating antibiotics within their pores [108,109]. Moreover, MSNs are relatively easy to functionalize, their surface modification improving the colloidal stability and targeting ability towards desired cells/tissues [13,110,111]. Nanoporous silica nanoparticles can also be used in treating inner ear diseases. They can target spiral ganglion neurons, being loaded with a brain-derived neurotrophic factor that is released in the long term [112].

Silicon carbide nanoparticles (SiC NPs) are inert antibacterial, hemocompatible, biocompatible, and non-toxic ceramic NPs that have been recently researched for biomedical applications [113,114]. SiC NPs were proven to enhance the antimicrobial activity of other materials when used as additives, showing improved in vitro results against *E. coli* and *S. aureus* [115]. Other ceramic nanoparticles of interest against inner ear-related pathogens are lithium niobate (LiNbO_3_) [1], silicon nitride (Si_3_N_4_) [116], titanium carbide (TiC) [117], and barium titanate (BaTiO_3_) [118].

### 4.2. Lipid Nanoparticles

Different lipid NPs have also been tested as delivery systems to the inner ear [77]. Formulations employing lipid core nanocapsules (LCNs), solid lipid NPs (SLNs), and phospholipid-based NPs are considered attractive due to their biodegradability and ability to deliver hydrophilic and/or lipophilic drugs [77,82].

Lipid core nanocapsules consist of a lipidic core made of triglycerides and mineral oils, with a surrounding shell of lecithin, polyethylene glycol, or poloxamers as stabilizing agents [13,55,119]. The LCNs’ structure can be modified to include various hydrophobic drugs and control their release kinetics [13,55]. Studies have shown promising results concerning LCNs permeation through the round window membrane and distribution throughout human inner ear cell populations [119], proving these particles’ potential in treating inner ear infections.

Solid lipid nanoparticles are also researched for delivering drugs to the inner ear [120]. SLNs are sub-micron colloidal carriers with unique properties, such as high drug loading and interaction of phases at the interfaces, which render them attractive for improving pharmaceuticals performance [121,122]. SLNs are reported to be a better alternative to liquid systems, as they form biocompatible and biodegradable lipids that are solid at body temperature, leading to improved control over drug delivery [77]. SLNs encapsulate the drug, improve its stability, and increase in vivo bioavailability, the delivery system exhibiting protective effects on the cochlea [123]. As a novel alternative to antibiotics, SLNs loaded with antibacterial oligonucleotide therapeutics have been investigated against *E. coli*, with promising results [124].

**Figure 5:**
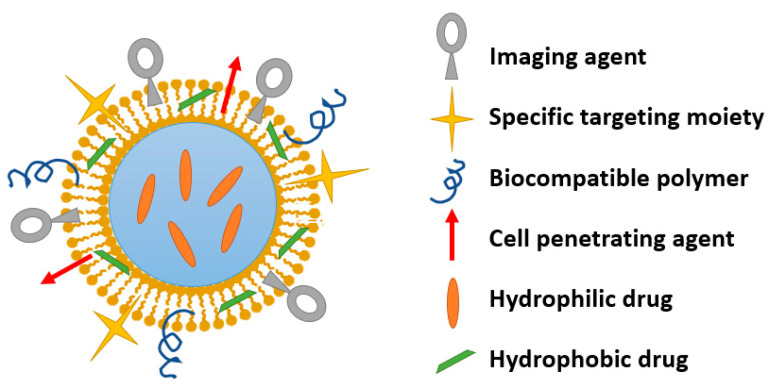
Schematic representation of a multifunctional nanoparticle. Created based on information from literature References [125,126,127,128].

Phospholipid-based NPs are advantageous structures as they can encapsulate hydrophobic molecules in their phospholipid layer and hydrophilic molecules in their aqueous core. Due to their similarity with plasma membranes, amphiphilic liposomes can transport their load across the round window membrane and deliver it inside the targeted cells [13,56,125]. Moreover, liposomes allow surface modification with various chemical and biological entities, such as polyethylene glycol, antibodies, peptides, carbohydrates, chitosan, hyaluronic acid, and folic acid, leading to multifunctional nanoparticles [13,55] (Figure 5).

### 4.3. Polymeric Nanoparticles

The variety and versatility of polymers have also attracted interest towards developing nanoplatforms for infection treatment. Several polymeric nanostructures have been shown effective as antimicrobials against ear-related pathogens, such as *E. coli*, *P. aeruginosa*, *S. aureus*, *K. pneumonia*, and *C. albicans* [129]. Nonetheless, polymers are most frequently studied as drug carriers.

One of the most extensively researched polymers is poly (lactic-co-glycolic acid) (PLGA), a Food and Drug Administration (FDA) and European Medicines Agency (EMA)-approved biodegradable copolymer that can encapsulate diverse molecules (e.g., proteins, steroids, antibiotics, nucleic acids) [55]. Due to their ability to adapt to specific requirements concerning drug properties and target tissue, PLGA NPs have a great potential in local inner ear delivery [55,56]. For instance, it has been demonstrated that rhodamine-loaded PLGA NPs can permeate through the round window membrane when applied locally, leading to a higher cochlear uptake than by systemic administration [56]. Moreover, functionalization with hydrophilic surface molecules has been proved to enhance permeability and successfully deliver rhodamine, SPIONs, and steroids in the inner ear, both in animal and human models [82].

Chitosan is another non-toxic, safe, and biodegradable polymer that can help increase the efficiency of inner ear disease treatment [79,125,130,131]. In addition to its antibacterial and antifungal activity [132,133], chitosan has a great potential in delivering therapeutics in a controlled and sustained manner from the middle ear to the inner ear without altering inner ear structures [134]. Besides, engineered fluorescence traceable chitosan NPs were recently shown to pass through the oval window into the vestibule. The successful experiment performed in guinea pigs opens the door for designing such delivery systems for treating peripheral vestibular diseases [82].

Other polymers and copolymers of interest for drug delivery include, but are not limited to, hyaluronic acid, poloxamer 407, poly (L-lactic acid) (PLLA), poly ε-caprolactone (PCL), polyethylene glycol (PEG), PLLA-PEG, and PLLA-PEG-PLLA [2,125].

A special class of polymeric NPs is represented by polymersomes. Moreover, called multifunctional NPs, polymersomes are amphiphilic copolymers that self-assemble into membranes of hydrophobic units around an aqueous core and a hydrophilic corona. The obtained structure resembles liposomes and has the advantage of controlling membrane thickness by the molecular weight of the copolymer’s hydrophobic block to achieve stronger, thicker, and more stable membranes [13]. Similar to liposome-based delivery systems, polymersomes can carry hydrophilic drugs in their core and hydrophobic ones in the membrane, the biomimetic structure enabling good immune tolerance [55]. Generally, polymersomes used in inner ear delivery consist of di-block copolymers (e.g., poly(ethylene glycol)-b-poly(ε-caprolactone)(PEG-b-PCL) or poly(2- hydroxyethyl aspartamide) (PHEA) [55]) that, at room temperature, form quite stable systems with the encapsulated drug [125]. In this respect, various multifunctional polymersomes were studied for inner ear drug delivery targeting specific tissue or conjugated with ferromagnetic materials [13].

### 4.4. Nanoparticles Incorporated in Nanocomposite Materials

Increasing scientific interest has also been observed in developing antimicrobial nanocomposite materials incorporating some of the above-described nanoparticles. In this regard, Banerjee et al. [135] have created an iodine-doped chitosan nanoparticle composite that proved synergic activity of the three antimicrobial components against *E. coli*, while minimizing AgNPs concentration and subsequent toxicity towards mammalian cells. Ziąbka et al. [88] proposed the incorporation of AgNPs into acrylonitrile butadiene styrene (ABS) polymer prosthesis to avoid infections (e.g., otitis media and chronic otitis media) in individuals requiring ossicular replacement prostheses.

Danti et al. [1] have focused their research on incorporating lithium niobate NPs into poly (vinylidene fluoride-trifluoro ethylene) fibers via electrospinning. The composite fibrous structure showed an enhanced piezoelectric response, supported human neural-like cell growth in vitro, and showed antibacterial activity against *P. aeruginosa*, being considered a promising candidate material for next-generation cochlear implants.

Another composite nanomaterial that may be of interest for drug delivery to the inner ear is represented by uniform magnetic spheres with magnetic core and mesoporous silica shell, developed by Zhao et al. [136]. The outer ceramic layer presents a high enough surface area and pore volume for encapsulating drugs, while the inner Fe_3_O_4_/Fe core endows this nanocomposite with magnetic properties, which are beneficial for carrying the drug to the targeted site. Namazi et al. [137] have also considered ceramic nanoparticles for creating a nanocomposite controlled-release system. The researchers fabricated an antibiotic-loaded hydroxymethylcellulose-MSNs composite hydrogel film intended for wound healing; however, its antibacterial activity against *S. aureus* can be useful in ear infections treatment as well.

### 4.5. Nanomaterials Safety

The studies in the field have also been focused on the safety of these nanomaterials, not only on treatment efficacy. In vitro and in vivo models were employed for assessing the effectiveness and potential side effects of several nanoparticles, focusing on parameters like cellular uptake, distribution in inner ear tissues, survival rates of treated cells, hearing threshold, and morphological changes after NPs administration.

In vitro tests are generally performed on cells from the House Ear Institute-Organ of Corti 1 (HEI-OC1) or Mouse Organ of Corti (OC-k3) cell lines, as they express many inner ear biomarkers [1,96]. Other models involve cells isolated from different inner ear structures of guinea pigs, rats, or mice cultured in situ [82,138,139,140]. Regardless of cell provenience, further experimental steps are similar. The most used method for measuring cellular metabolism as an indicator of cell viability, proliferation, and cytotoxicity is the 3-(4,5-dimethylthiazol-2-yl)-2,5-diphenyltetrazolium bromide (MTT) assay. It assumes the seeding onto a 96-well plate of cells exposed to different concentrations of NPs, followed by 24 h incubation. Then, the culture medium is discarded, cells are rinsed twice with phosphate-buffered saline, and MTT solution is added. After four more hours of incubation, cells are solubilized with dimethyl sulfoxide, and the plate is left on a shaker in the dark for 2 h. In the end, the absorbance is measured at 570 nm and compared with control samples [96,141]. Similarly, cell viability can be determined by the CCK-8 (cell counting kit-8) assay, the main difference consisting of monitoring the absorbance at a different wavelength (i.e., 450 nm) [142]. Another colorimetric method for investigating cytotoxicity is the 3-(4,5-dimethylthiazol2-yl)-5-(3-carboxymethoxyphenyl)-2-(4-sulfophenyl)-2H-tetrazolium (MTS) assay in which cells are seeded to the 96-well plate, left 24 h to adhere, then treated with the NPs suspended in a complete medium at various concentrations. Cells are analyzed at 24, 48, and 72 h after treatment; at the end of each time point, the cells’ medium is changed with complete medium plus MTS-phenazine methosulfate mixture and left for three more hours to incubate. In the end, the absorbance is read at 492 nm and normalized to the average value of untreated samples [1].

In what concerns in vivo studies, they usually imply NPs administration to anesthetized animals by intratympanic/trans-tympanic injection, followed by different tests depending on what is desired to be measured. Experiments may be performed on alive laboratory animals if the auditory function has to be tested, or, at a certain time after treatment administration, the animals are decapitated under deep anesthesia, and the tissues of interest are carefully explanted for further analyses.

Table 1 summarizes several identified in vitro and in vivo studies investigating the potential adverse effects of nanoparticles on inner ear cells.

Zhang et al. [138] demonstrated that an increase in LCNs concentration leads to a decrease in the in vitro viability of the inner ear cochlea cells. Nonetheless, LCNs with sizes below 100 nm are biodegradable, and their in vivo administration does not produce any infection, inflammation, hearing impairment, cell death, or morphological changes in the inner ear. Zhou et al. [139] reported a time-dependent decrease in cell viability of mice cultured cochlear epithelium treated with linear PEI-plasmid DNA-NPs, and several concerns are to be solved before in vivo testing (e.g., PEI limited biodegradability, high cationic charge density, production of intracellular reactive oxygen species). An ototoxicity-size dependency was observed in vitro by Nguyen et al. [140]. However, in vivo administration of SPIONs of 200 nm has shown no difference in hearing threshold compared to saline-treated ears, rendering these NPs as promising vectors for controlled delivery to cochlear targeted cells. Musazzi et al. [143] have registered increasing cell death rates with increasing doses of resveratrol-loaded PLGA NPs. Particularly, cell viability decreased in HEI-OC1 cells exposed to high concentrations of the tested NPs, while SVK-1 cells proved to be more resistant to NPs exposure; nonetheless, future experiments are needed for deeply investigating cellular uptake mechanisms and intracellular release of the loaded drug. PLGA NPs were also evaluated by Wen et al. [142]. The researchers tested different surface-modified polymer NPs, concluding that biocompatible PLGA-based nanocarriers, if functionalized with hydrophilic molecules, have a greater capacity to penetrate outer hair cells, thus allowing a more efficient hearing loss therapy. No significant toxicity, no observable changes in cell viability, cell morphology, or auditory brainstem response were reported for AuNPs [96], chitosan NPs [130], methoxy poly (ethylene glycol)-polylactic acid nanoparticles loaded with dexamethasone [144], lipid nanoparticles-encapsulated brain-derived neurotrophic factor mRNA [145], and lithium niobate NPs [1].

In conclusion, a general observation that can be drawn from these studies is that ototoxicity is highly dependent on nanoparticle size and concentration, while NPs functionalization dictates distribution into targeted tissues and uptake efficiency. Hence, these factors must be thoroughly considered when designing nanoparticles-based treatments for the inner ear.

## 5. Conclusions and Future Perspectives

To summarize, the inner ear is sensitive to various bacterial, viral, and fungal infections, which may produce permanent sensorineural hearing loss. If left untreated, such infections can progress to neighboring tissues and cause intracranial infectious complications. Therefore, prompt treatment is required. The necessary medication can be administered in several ways, such as systemic delivery, intratympanic injection, and direct inner ear delivery. However, each method has limitations in overcoming the inner ear barriers.

A promising solution for an efficient and targeted treatment of inner ear infections comes from the field of nanotechnology. Different types of nanoparticles were designed and tested either directly against pathogens or as carriers of various drugs. Several inorganic, lipid and polymeric-based nanoplatforms were shown to improve drugs’ local application, enhancing antimicrobial performance while diminishing the systemic side effects. Studies were also performed on the safety of nanoparticles’ use for inner ear delivery, demonstrating that most of these materials are harmless for healthy tissues.

However, there is a lack of information concerning nanomaterials ear toxicity in human beings, possible adverse effects in organ systems which are not generally considered primary ototoxic targets, and long-term impact on ear health. Hence, future studies must also consider elucidating these aspects of nanoparticle-mediated treatments.

A promising future perspective for treating inner ear infections would be the development of biocompatible multifunctional nanoparticles capable of targeting specific cells, deliver drugs in a controlled manner, and biodegrade into harmless entities that can be easily eliminated from the organism. Alternatively, multifunctionality should be sought by designing synergic composite nanomaterials.

To conclude, current research results indicate the great potential of nanoparticles in treating various inner ear diseases. It can be expected that certain nanomaterials or nano-enabled products would soon be available as therapeutic options. Nonetheless, more in vivo studies must be carried out before moving to clinical applications.

## Figures and Tables

**Table 1:** Summary of studies on nanomaterials potential side effects on inner ear cells.

Tested Nanomaterial	Nanomaterial Properties	Type of Study	Type of Cells	Experimental Design	Observations	Refs.
LCNs	Size: 50 nm Polydispersity index: <0.2	In vitro	Cochlear cells isolated from newborn Sprague-Dawley rats	The cells were treated with LNCs at concentrations varying between 0 and 1.5 mg/mL for 24 h.	Survival rates of treated cells, depending on concentration: −1.5 mg/mL—37.96% −0.15 mg/mL—86.41% −0.015 mg/mL—80.06% Surviving cells from all treated groups had fewer LNCs in their cytoplasm.	[82,138]
LCNs	Nanoparticle size: 50 nm Pledget size: 8 mm^3^ LNC concentration: 20.5 g/L	In vivo	Interdental cells, stria marginal cells, outer hair cells, inner hair cells, semi-circular canal endothelial cells, cochlear nerve of rats	A small piece of gelatin sponge pledget saturated with LNCs was placed on the round window membrane, and it was there for D28 for ABR study and 2 h for neural elements studies.	None of the animals manifested middle ear infections during the study. No inflammation was detected in the inner ear LNC treatment did not induce apoptosis. The inner ear neural elements were preserved.	[138]
Resveratrol-loaded PLGA nanoparticles	Size: 135.5 ± 37.3 nm	In vitro	HEI-OC1 and SVK-1 cell line	The cells were treated with blank nanoparticle, resveratrol, and resveratrol-loaded nanoparticles at concentrations up to 1 mg/mL for 24 h.	No cell line’s viability was affected by blank nanoparticles in concentrations below 0.6 mg/mL At a concentration of 1 mg/mL, blank nanoparticles produced the death of 56% cells from HEI-OC1 cell line Resveratrol and resveratrol-loaded nanoparticles lead to negligible cell death rates.	[82,143]
Polyethylenimine (PEI)-plasmid DNA nanoparticles	Size: ~20–100 nm Shape: almost spherical	In vitro	Cochlear epithelium isolated from C57BL/6J male and female mice	The cochlear explants were treated with linear nanoparticle polyplexes loaded with plasmid DNA at various weight ratios; the cell viability was assessed in a 0–48 h interval after transfection.	The use of a higher linear polyethylenimine-plasmid DNA ratio conducted to a significant time-dependent reduction in hair cell viability Oto-nanotoxicity of the tested material started to manifest immediately after the addition of the poly-plex, especially outer hair cells were noted to be more vulnerable in the acute phase.	[82,139]
SPIONs	Size: 100 and 500 nm	In vitro	EC5V cells derived from the inner ear ampulla of semicircular canals.	The cells were treated with SPIONs at final concentrations, depending on size: 100 nm—3 × 10^10^, 3 × 10^9^, 3 × 10^8^ NP/mL 500 nm—7 × 10^7^, 7 × 10^6^ NP/mL.	A lower number of surviving cells were reported in the 100 nm treated group than in the 500 nm and control groupsApoptotic cells were more frequently observed in the 100 nm group than in the 500 nm and control groups.	[82,140]
SPIONs	Size: 200 nm	In vivo	Inner ear cells of albino male guinea pigs	In each animal, on one ear, a 0.4 mm scala tympani cochleostomy, 1.5 mm under the round window ridge was performed through a posterior approach and bullostomy and 1 μL of saline serum was injected. In the other ear, a bolus of 1 μL of nanoparticles was performed using the same method.	At day 7, hearing threshold shift showed no difference between saline-treated ears and nanoparticles treated ears.	[140]
AuNPs	Size: 50 nm Shape: spherical	In vitro	HEI-OC1 cell line	The cells were treated with nanoparticles at 0–100 μM for up to 6 days	There were not reported any significant changes in cell viability.	[82,96]
AuNPs	Size: 50 nm Shape: spherical	In vivo	Mouse cochlear cells	Gold nanoparticles were applied in vivo to mouse cochleae	The injected nanoparticles fully diffused throughout the inner ear and were successfully localized within the cells. The presence of nanoparticles had no observable effect on the morphology of the hair cells. Gold nanoparticles do not enhance X-ray attenuation in a significant manner. Hence they are not considered suitable computed tomography imaging contrast agents for the inner ear.	[96]
Methoxy poly (ethylene glycol)-polylactic acid nanoparticles loaded with dexamethasone	Size: 130 nm Shape: spherical	In vivo	Inner ear cells of male guinea pigs	The treatment was administered intraperitoneally at a dose of 10 mg/kg and at a concentration of 10 mg/mL, 1 h before cisplatin injection. Three days after treatment, the animals were euthanized, and their tissues were prepared for the examination.	The auditory brainstem response threshold was not significantly changed, indicating nanoparticles’ nontoxicity. A single injection of nanoparticles was reported to provide significant functional and histological protection of the cochlea from the cisplatin, which was similar to the effect of repeated injection of the free drug for 3 days.	[144]
Unmodified PLGA-nanoparticles, surface modified with poloxamer 407, chitosan, or methoxy poly(ethylene glycol)	Size: 100–200 nm Relatively uniform size distribution	In vitro	HEI-OC1 cell line	The cells were treated with nanoparticles at concentrations varying between 0 and 80 mg/mL for 24 h	IC_50_ values: (1) unmodified NPs—71.30 ± 4.16 mg/mL (2) P407-modified NPs—60.53 ± 0.55 mg/mL (3) Chitosan-modified NPs—65.39 ± 0.47 mg/mL (4) mPEG-modified NPs—81.70 ± 1.04 mg/mL Uptake efficiencies: (1) unmodified NPs—79.7% (2) P407-modified NPs—91.4% (3) Chitosan-modified NPs—58.3% (4) mPEG-modified NPs—48.1%	[82,142]
Unmodified PLGA-nanoparticles, surface modified with poloxamer 407, chitosan, or methoxypoly (ethylene glycol)	Size: 100–200 nm Relatively uniform size distribution	In vivo	Inner ear cells of albino guinea pigs	The four types of nanoparticles were injected at a concentration of 25 mg/mL into the unilateral tympanic cavity of the guinea pigs. They were examined 24 h after administration	No inflammation was detected in the inner ear Several tissues, such as stria vascularis, spiral ligament, organ of Corti, and spiral ganglion cells, barely underwent morphological alterations after nanoparticles administration The hydrophilic coating of PLGA nanoparticles played an important role in inner ear transport, particularly the P407 modification The surface-modified nanoparticles were considerably localized in the spiral ligament, stria vascularis, organ of Corti, and spiral ganglion cells, while the unmodified particles were distributed marginally	[142]
Chitosan nanoparticles	Average size: 152.7 nm Polydispersity index: 0.135 Shape: spherical	In vitro	HEI-OC1 cell line	The cells were treated with nanoparticles at concentrations varying between 0 and 2.5 mg/mL for 24 h	There was not reported any significant change in cell viability Nanoparticles were internalized in cells with an 89.1% uptake efficiency	[82,130]
Chitosan nanoparticles	Average size: 152.7 nm Polydispersity index: 0.135 Shape: spherical	In vivo	Inner ear cells of guinea pigs	The nanoparticles were injected at a concentration of 2.5 mg/mL into the unilateral tympanic cavity of the guinea pigs. The animals were decapitated 1 h after the treatment and examined after 24 h	The number of surviving hair cells hardly decreased, indicating the safety of the tested nanoparticles. The chitosan nanoparticles were successfully delivered into the vestibule, accumulating significantly more in the vestibular system than in the cochlear tissues The nanoparticle uptake in saccular supporting cells was much higher compared to cochlear hair cells of middle and apical turns	[130]
Lipid nanoparticles-encapsulated brain-derived neurotrophic factor (BDNF) mRNA	Lipid composition: SS-cleavable and pH-activated lipid-like material:dioleolyphosphatidyl ethanolamine (DOPE):cholesterol= 3:3:4	In vivo	Inner ear cells of Hartley guinea pigs	The animals were intramuscularly injected with gentamicin, promptly followed by intravenous injection of ethacrynic acid. On day 1, for early therapy, or day 14, for late therapy, 5 μl of lipid nanoparticles loaded with 0.1 mg/mL BDNF -enhanced green fluorescent protein mRNA was administered	On day 1 after gentamicin exposure, the auditory thresholds of the group administered with nanoparticles significantly improved compared to the sham control group. The auditory thresholds did not differ significantly between the sham control group and animals administered with nanoparticles 14 days after gentamicin exposure. The outer hair cells in the cochlea of the group treated 1 day after gentamicin exposure were significantly decreased compared with those in the control group, while inner hair cells counts had no significant differences in all turns among all groups.	[145]
Lithium niobate NPs	Size range: 200–600 nm Average size: 392.25 nm Polydispersity index: 0.517	In vitro	OC-k3 cell line	(1) Cytotoxicity of nanoparticles was investigated using the MTS assay. The OC-k3 cells were seeded in 96-well plates at the concentration of 7000 cells/well in 100 μL of medium and left to adhere for 24 h at ambient temperature after which were treated with the compound resuspended in a complete medium at three different concentrations: 0.85, 15, and 74 ng/mL. Vitality was analyzed 24, 48, and 72 h after treatment. (2) Similarly, a morphological test was performed by seeding and culturing the cells on a round glass slide.	The tested nanoparticles induced a significant increase in cell viability after 72 h of incubation at the concentrations of 0.0085 and 0.015 μg/mL. The morphological test proved a good state of cellular health. No cell morphology alterations were noticed at any of the tested doses and time points.	[1]
